# Experimental Investigation of Material Properties and Self-Healing Ability in a Blended Cement Mortar with Blast Furnace Slag

**DOI:** 10.3390/ma13112564

**Published:** 2020-06-04

**Authors:** Seunghyun Na, Wenyan Zhang, Madoka Taniguchi, Nguyen Xuan Quy, Yukio Hama

**Affiliations:** 1 Institute of Industrial Science, The University of Tokyo, 4-6-1 Komaba, Meguro-ku, Tokyo 153-8505, Japan; nash1122@naver.com; 2 School of Material Science and Engineering, Henan Polytechnic University, Jiaozuo 454-000, China; zhangwy@hpu.edu.cn; 3 Environmental Engineering Division, Northern Regional Building Research Institute, Hokkaido Research Organization, Asahikawa, Hokkaido 078-8801, Japan; madoka@hro.or.jp; 4 College of Environmental Technology, Graduate School of Engineering, Muroran Institute of Technology, Muroran 050-8585, Japan; quynx@hau.edu.vn; 5 Department of Civil Engineering, Hanoi Architectural University, Hanoi 100000, Vietnam

**Keywords:** frost resistance, self-healing ability, blast furnace slag, freeze–thaw cycles, relative dynamic modulus of elasticity, carbonation coefficient

## Abstract

This paper presents the results of an experimental investigation on the material properties and self-healing ability of a blended cement mortar incorporating blast furnace slag (BFS). The effect of different types and Blaine fineness of BFS on the material properties and self-healing was investigated. Thirteen cement mixtures with BFS of different types and degrees of Blaine fineness are tested to evaluate the mechanical properties, namely compressive strength, bending strength, freeze–thaw, and accelerated carbonation. The pore structure is examined by means of mercury intrusion porosimetry. Seven blended mortar mixtures incorporating BFS for cement are used to evaluate the mechanical properties after applying freeze–thaw cycles until the relative dynamic modulus of elasticity reached 60%. The experimental results reveal that incorporating BFS improves the mechanical properties and self-healing ability. In the investigation of self-healing, smaller particle and high replacement ratios of BFS contribute to increasing the relative dynamic modulus of elasticity and decreasing the carbonation coefficient in the mortar after re-water curing. Moreover, BFS’s larger particles and high replacement ratio are found to provide better self-healing ability. A regression equation is created to predict the relative dynamic modulus of elasticity in mortar considering the Blaine fineness, BFS replacement ratio, and curing conditions.

## 1. Introduction

Concrete has been widely used for a long time in the civil engineering and construction fields, and it has many benefits, such as high durability, low cost, and high compressive strength. However, although there are many advantages, the damage of a concrete structure is inevitable. In cold environments, the deterioration of a concrete structure depends on not only the moisture content but also the weather conditions, such as the freezing temperature and the number of freeze–thaw cycles in the literature [1,2]. Kamada pointed out that the freeze–thaw resistance depends on pore structure in the concrete, pore volume of 40–2000 nm in diameter [3]. The deterioration of concrete by frost damage may decrease the compressive strength, bending strength, and carbonation resistance. Moreover, the decreasing performance due to micro cracks generated by freeze–thaw cycles reduces the lifetime of the concrete structure. Therefore, it is necessary to develop a technique capable of healing such cracks.

Up to now, many techniques have proposed to increase frost resistance in concrete. It is generally known that mixture design optimization for the reduction of water content to form much denser microstructure, increasing the air content, and decreasing the air void spacing factor are some of the successful techniques [4]. According to recent studies, Fujii and Ayano investigated the effect of the addition of blast furnace slag (BFS) on the frost resistance of concrete without air entrain agent and reported that freeze–thaw resistance of concrete increases when BFS and fly ash were used with fine aggregate [5]. However, the effect of the type of BFS on the freeze–thaw resistance of cement-based materials was not examined.

Consequently, there has been research into self-healing abilities as a promising solution to the issue of damaged concrete. It has been reported that self-healing was classified into natural healing, autonomic healing, activated healing according to the Japan Concrete Institute [6]. Natural healing is re-hydration of residual unhydrated cement with low water to cement ratio or re-hydration cracks found in hydraulic structures.

Furthermore, various materials for autonomic healing have been used, such as expansive agent [7,8], fly ash [9,10,11], bacteria [12,13], blast-furnace slag (BFS) [14,15,16,17], chemical admixtures [8], and engineered cementitious composite with fly ash, described by [6]. It reported that the fly ash has the long-term pozzolanic reaction and fills microcracks due to hydration products of fly ash [9]. Ahn et al. investigated the self-healing behavior of cement-based materials with mineral admixtures and reported that a crack of 0.15 mm was self-healed after three days of re-curing [8]. It was found that the phenomenon is due to the swelling effect, expansion effect, and re-crystallization of used materials. This study focused on the blocking water leakage. Sisomphon and Copuroglu reported that ettringite formation generates in the cracks [18]. In addition, the researchers have studied the self-healing ability of cement-based materials that are damaged by freeze–thaw. Studies have been performed to overcome the abovementioned problems and to investigate self-healing of cracks in cement mixtures incorporating fly ash [9,10,11]. It was shown to be possible to heal micro cracks in a cement mixture after frost damage. Incorporating fly ash in the mortar was reported to lead to not only an increase in the relative dynamic modulus of elasticity but also a decrease in the carbonation coefficient, and it was reported that cracks occurring after frost damage are filled by hydration products of the fly ash [12]. Therefore, it was found that the addition of fly ash seemed to be beneficial for filling cracks.

On the other hand, research on the effective use of BFS in cement-based materials have been performed to reduce the cracks [15]. In the self-healing of the addition of BFS on self-healing behavior, it can be concluded that self-healing after damage depends on the high percentage of cementitious material and smaller micro cracks that can be filled by unhydrated cement and BFS [14]. The main hydration products were C–S–H gels from the hydration of BFS, thus high recovery with respect to compressive strength can be achieved [17]. The use of BFS as a self-healing material has many advantages such as reducing carbon dioxide emissions and reducing manufacturing cost, compared to an expansive agent. However, there is a lack of information about the self-healing ability of blended cement mixtures for different types and Blaine fineness of BFS and cements by taking into consideration freeze–thaw cracks.

Therefore, the purpose of this study is to investigate the material properties and self-healing ability of blended cement mortar incorporating BFS of different types and degrees of Blaine fineness. Tests were carried out on the mortar samples to evaluate the mechanical properties, namely the compressive strength, bending strength, freeze–thaw, and accelerated carbonation, and the pore structure was investigated. Seven blended mortar mixtures incorporating BFS for cement were used to evaluate the mechanical properties after applying freeze–thaw cycles until the relative dynamic modulus of elasticity reached 60%. Then, a regression equation was created to predict the relative dynamic modulus of elasticity in mortar considering the Blaine fineness, BFS replacement ratio, and curing conditions.

## 2. Experiment

### 2.1. Materials

BFS and cement were used as binder materials in this study. Table 1 shows the chemical composition and physical properties of cement and BFS. The experiments used ordinary Portland cement (Nippon steel cement corporation, Muroran, Japan) N; specific gravity: 3.16 g/cm^3^; Blaine fineness: 3250 cm^2^/g). Three types of BFS were used, with fineness values of 3180, 4030, and 7300 cm^2^/g and densities of 2.91, 2.91, and 2.90 g/cm^3^, respectively. Natural land sand was used as the fine aggregate. The surface-dry density and the water absorption rates of the sand were 2.65 g/cm^3^ and 0.42%, respectively. The fineness modulus, surface-dry density, and the water absorption of the sand were 2.74, 2.65 g/cm^3^, and 0.42%, respectively.

### 2.2. Mixture Proportions and Specimen Preparation

The mixture proportions of the mortars are shown in Table 2. The three types of BFS were added to the mortar at replacement levels of 45% and 70% by weight of cement and 20% and 45% by weight of sand. The water-to-cement ratio (W/C = water/cement) was 0.55 and the water-to-binder ratios (W/B = water/(cement + BFS)) were 0.38, 0.45, and 0.55 for the three types, respectively. The flow value and air content of all the investigated mortars with and without BFS were examined in accordance with JIS R 5201 and JIS A 1171 [19,20]. Then, they were mixed and cast as 40 mm × 40 mm × 160 mm prisms to form the mortar test specimens. After 1 day, the samples were de-molded and cured at 20 °C in water for 28 days.

### 2.3. Testing Methods

Table 3 and Table 4 list the test items for all the samples used in this study. As shown in Table 3, the BFS-blended mortar samples were examined for their material properties, namely the compressive strength, bending strength, accelerated carbonation, and freeze–thaw. Two replicates of each of the mixtures for tests. Then, as shown in Table 4, seven mixtures were tested to evaluate the self-healing ability of the mortars incorporating BFS. Details of the test methods are presented below.

#### 2.3.1. Compressive Strength and Bending Strength Tests

The compressive strength and bending strength of the mortar test specimens were tested in accordance with JIS R 5201 [19].

#### 2.3.2. Accelerated Carbonation Depth

The accelerated carbonation test was conducted according to JIS A 1153. All of the mortar samples were cured to water during 28 days in water before testing [21]. Then, the samples were dried in a control chamber at 20 °C and 60% relative humidity for 28 days. Afterward, the samples were dried and the tests conducted with 5% carbon dioxide gas concentration at 1, 4, and 13 weeks, when the carbonation depth was measured using the phenolphthalein test. The carbonation coefficient was calculated from the carbonation depths measured from the investigated samples using the following equation:

(1) $$x=k\sqrt{t}$$

where *x* is the depth of carbonation (mm), *t* is the duration of carbonation (weeks), and *k* is the carbonation coefficient (mm·week^−0.5^).

#### 2.3.3. Freeze–Thaw Test

Freeze–thaw testing was performed for 300 cycles in accordance with JIS A 1148 [22]. The relative dynamic modulus of elasticity was determined by measuring the resonance frequency and was calculated as the percentage fraction of the square transverse frequency after the freeze–thaw test to the square transverse frequency before the freeze–thaw test, in accordance with JIS A 1127 [23] and the durability factor was calculated from the relative dynamic modulus of elasticity, using following equation.

(2) $$Pn=\left(\frac{{f_{n}}^{2}}{{f_{0}}^{2}}\right)\times 100\%$$

where *Pn* is the relative dynamic modulus of elasticity (%), *f_n_* is the fundamental transverse frequency at n ycle (Hz), and *f*_0_ is the fundamental transverse frequency at 0 cycles (Hz).

The durability factor was calculated from the results of freeze/thaw test of all investigated samples, as shown Equation (3):

(3) $$DF=\frac{P\times N}{M}$$

where *DF* is the durability factor of the test specimen; *P* is the relative dynamic modulus of elasticity at cycles (%); *N* is the number of cycles at which *P* reaches the specified minimum value less than 60% or the relative dynamic modulus of elasticity after 300 cycles, and *M* is the relative dynamic modulus of elasticity after 300 cycles.

#### 2.3.4. Mercury Intrusion Porosimetry

The pore structure was investigated via mercury intrusion porosimetry (MIP) by means of an Autopore Master 33 porosimeter (Quantachrome instruments, Anton paar Quanta Tec. Ltd., Tokyo, Japan) to evaluate the pore size distribution in the mortar samples. Pressures ranged 0–220 MPa. The surface tension and the density of mercury were 0.480 N/m and 13.546 g/mL, respectively. The contact angle of mercury was 140°. The mortar prisms after 28 days in water at 20° were cut into 5-mm-wide cube-shaped samples and then dried to stop the cement hydration reaction with acetone, according to D-drying pretreatment [24,25].

### 2.4. Investigation of Self-Healing Ability

To generate micro cracks in the samples, the mortar samples after 28 days of water curing underwent freeze–thaw testing until the relative dynamic modulus of elasticity was 60%. The relative dynamic modulus of elasticity was determined by measuring the resonance frequency and calculated from the result. After deterioration of the samples, different curing conditions were applied: 20 °C for 1 week to reflect curing conditions over 1 year and 40 °C for 4 weeks in water to investigate the self-healing ability, as reported in a previous study [9]. Then, tests to measure the physical properties, namely the resonance frequency, bending strength, compressive strength, accelerated carbonation depth, of the samples at different testing ages were performed. Moreover, accelerated carbonation samples after re-curing was dried to in a control chamber at 20 °C and 60% relative humidity for 28 days. Then, carbonation depths were measured according to Section 2.3.2. Moreover, general regression analysis was performed to predict the relative dynamic modulus of elasticity of the mortar samples, considering Blaine fineness, replacement ratio, and water curing conditions, using Minitab software (Version 17, Minitab Inc., Seoul, Korea).

## 3. Results and Discussion

### 3.1. Materials Properties

#### 3.1.1. Bending Strength

**Figure 1:**
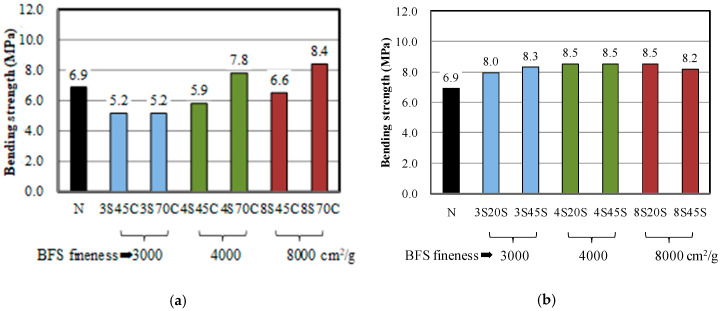
Bending strengths of mixtures made with different BFS replacement ratios: (**a**) BFS as a replacement for cement and (**b**) BFS as a replacement for sand.

Figure 1a,b show the results of bending strength and compressive strength tests for BFS-blended cement mortar samples with different Blaine fineness values. The BFS replacement ratios were set at 45 wt % and 70 wt % for cement, and 20 wt % and 45 wt % for sand in the mortar. Thirteen mixtures were prepared with the proportions summarized in Table 2. The bending strength of mortar sample N was approximately 7 MPa. For BFS as a replacement for cement, as shown in Figure 1a, it can be seen that mortar samples 3C45 and 3C70 produced with a low fineness of 3000 cm^2^/g have a lower bending strength than that of sample N, which is the mortar without BFS. However, the bending strengths of mortar samples 4C70C and 8C70C are higher than those of the other mortar samples, implying that high replacement ratios and BFS fineness values had a positive effect on the bending strength of the mortar.

For BFS as a replacement for sand, as shown in Figure 1b, the bending strengths of all the mortar samples made with BFS were higher than that of mortar N. These BFS-blended mortars have bending strengths over 8 MPa, even though the BFS fineness values and replacement ratios were different. This indicates that incorporating BFS for sand can contribute to the increase in the bending strength in mortar.

#### 3.1.2. Compressive Strength

**Figure 2:**
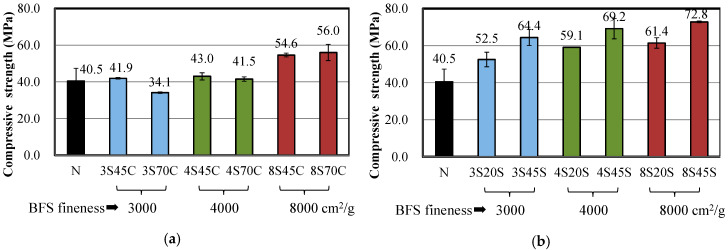
Compressive strengths of mixtures made with different BFS replacement ratios: (**a**) BFS as a replacement for cement and (**b**) BFS as a replacement for sand.

Figure 2a,b present the results of compressive strength tests for the mortar samples. As shown in Figure 2a, the compressive strength of mortar samples 8C45 and 8C70 with high Blaine fineness values of 8000 cm^2^/g was considerably higher than that of mortar N, implying that the addition of BFS as a replacement for cement with high Blaine fineness is effective for the development of compressive strength in BFS-blended mortar. As shown in Figure 2b, when using BFS as a replacement for sand, all mortar samples with BFS were found to have higher compressive strength compared to mortar N, which also agrees with the results of the bending strength test in Section 3.1.1. Moreover, in the case of BFS as a replacement for sand, a low W/C may cause an increase of compressive strength, owing to the decrease of the total pore volume with respect to the MIP results [26].

#### 3.1.3. Mercury Intrusion Porosimetry

**Figure 3:**
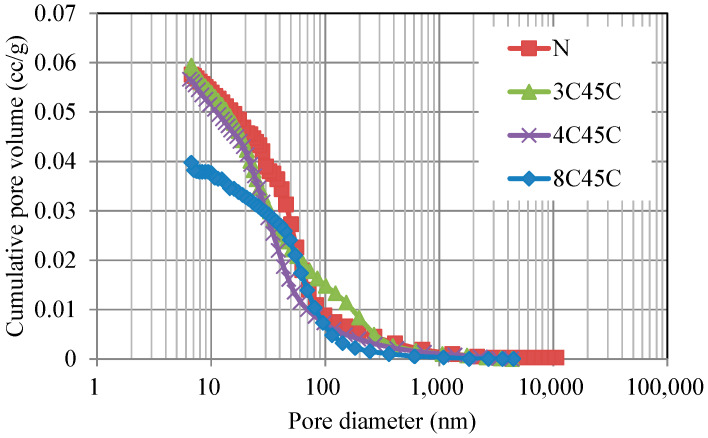
Cumulative pore volume of BFS-blended mortar with different Blaine fineness values for BFS by means of mercury intrusion porosimetry.

The MIP results are presented in Figure 3. The total pore volumes of mortars N, 3C45, 4C45, and 8C45 were found to be 0.0576, 0.0594, 0.0567, and 0.0398 cc/g, respectively. It can be seen from the figure that the pore volume of mortar 8C45 was smaller than that of the other mortar samples, which is due to the densification of the smaller fineness and the hydration of BFS that can fill the pore volume in the BFS-blended mortar sample. Therefore, as mentioned above for Figure 2a, the increasing compressive strength of 8C45 in comparison to mortar N is due to the smaller pore volume in the BFS-blended mortar sample, which is similar to the results of a previous study [27].

#### 3.1.4. Freeze-Thaw Test

In general, as the number of freeze–thaw cycles increases, the decrease of the relative dynamic modulus may cause an increase in the number of cracks within the mortar sample because of internal cracking in the cement. This causes not only the increased weight of the mortar sample but also the increased length change compared to the initial state. Moreover, the relative dynamic modulus of elasticity was approximately 60% in the mortar, which is considered to be poor performance for frost resistance [28].

**Figure 4:**
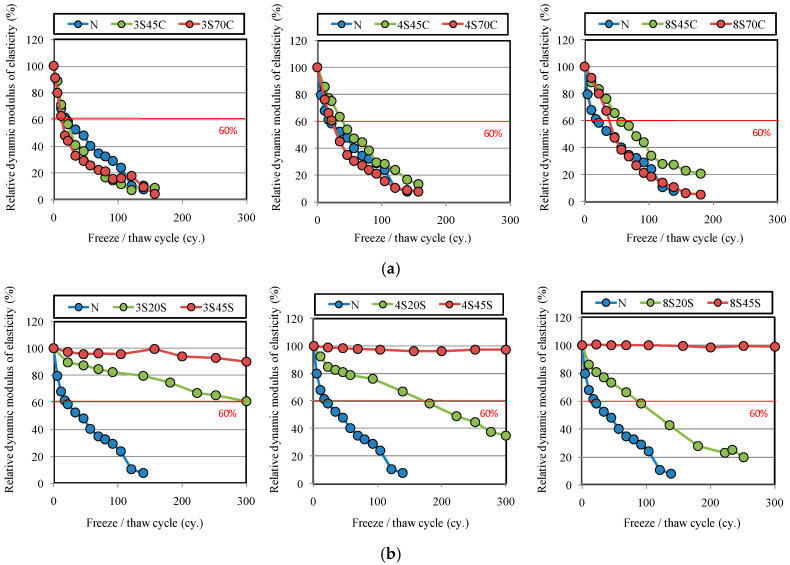
Relative dynamic modulus of elasticity for different mortar mixtures with or without BFS during freeze–thaw cycles: (**a**) replacement by BFS for cement and (**b**) replacement by BFS for sand.

Figure 4a,b present the results of freeze–thaw tests of the mortar samples. As can be seen from Figure 4a, the relative dynamic modulus of elasticity for all mortar samples decreased below 60% before 100 cycles. Therefore, an air-entraining agent is needed to prevent frost damage in the mortar samples.

However, it is clear from Figure 4b that the relative dynamic modulus of elasticity of the mortar samples with 20 or 45 wt % of BFS replacement for sand does not decrease as the number of freeze–thaw cycles increases, unlike mortar N. Moreover, with increasing BFS replacement ratio, a high relative dynamic modulus of elasticity of 90–100% at 300 cycles was observed for the mortar samples, especially for 3S45S, 4S45S, and 8S45S. These observations indicate that an increase in BFS replacement for sand yielded significantly better frost resistance performance in BFS-blended mortar. This result is similar to the study by Ayano and Fugii [5].

Moreover, in the case of 45% of BFS with sand, effect of Blaine fineness of BFS on frost resistance was not significant, which implies that frost resistance in the BFS blended mortar depends on the ratios of BFS replacement for sand.

**Figure 5:**
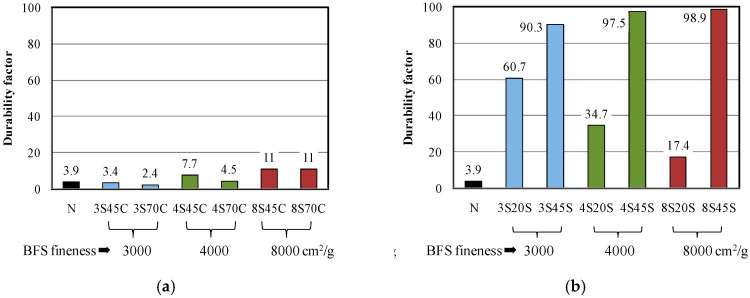
Durability factors for different mortar mixtures with or without BFS during freeze–thaw cycles: (**a**) replacement by BFS for cement and (**b**) replacement by BFS for sand.

The durability factor was calculated from the results of the relative dynamic modulus of elasticity of each mixture and the results are shown in Figure 5. The durability factor of the sample with BFS as a replacement for sand is significantly higher than that of the other mortar samples. Furthermore, in the case of a BFS replacement ratio of 45 wt %, the durability factor was 90–100, implying that BFS replacement for sand may improve the frost resistance of the BFS-blended mortar samples, which is related to the higher compressive strength before the freeze–thaw test.

#### 3.1.5. Accelerated Carbonation Coefficient

**Figure 6:**
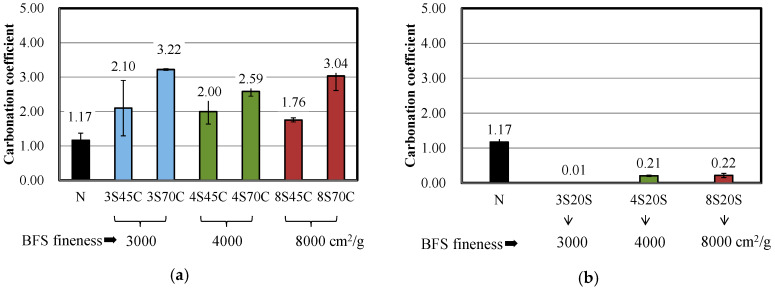
Carbonation coefficients for different mortar mixtures with and without BFS: (**a**) replacement of BFS for cement and (**b**) replacement of BFS for sand.

Figure 6 presents the results for the carbonation coefficients of the mortar samples with and without BFS. The carbonation coefficient of mortar N was 1.17. The carbonation coefficient tends to increase with the BFS replacement ratio over that of mortar N. This is due to the fact that Ca(OH)_2_ in the mortar incorporating BFS with cement is more consumed, which causes the carbonation process to be accelerated. However, in the case of BFS replacement for sand, the carbonation coefficient of the mortars was significantly lower than that of mortar N, implying that replacement of the slag for sand in the BFS mortar samples is advantageous for the improvement carbonation resistance. Yoshida and Saito reported that the concrete sample with BFS decreased the air permeability, which is related to the transition zone around the particle of BFS due to the hydration of BFS with increasing age [29]. Therefore, as shown in Figure 6b, it is considered that the carbonation coefficient is reduced because the matrices of the mortar with mixed BFS were densified, thus, invasion of CO_2_ ions in inhibited by the hydration reaction of slag.

### 3.2. Self-Healing Effect

#### 3.2.1. Investigation of Degree of Damage

**Figure 7:**
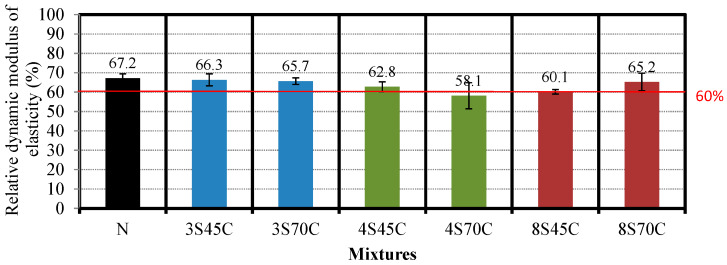
Results for the relative dynamic modulus of elasticity of the different mixtures after damage.

The freeze–thaw test was performed until a relative dynamic modulus of elasticity of 60% was reached, in accordance with JIS A 1127 [23], in order to generate cracks in the mortar sample. Figure 7 presents the results for the relative dynamic modulus of elasticity for BFS-blended cement mortar samples. The results for the relative dynamic modulus of elasticity of all the mortar samples range between 58.1% and 67.2%, and were approximately 60%, implying that these mortar samples satisfied the target relative dynamic modulus of elasticity. Then, after being damaged, the mortar samples were stored under two types of curing conditions, namely curing in water at 40 °C for 4 weeks and in water at 20 °C for 1 week [9]. After that, the compressive strength, bending strength, accelerated carbonation depth, and relative dynamic modulus of elasticity of these mortar samples were examined.

#### 3.2.2. Bending Strength, Compressive Strength, and Carbonation Coefficient of Mortar Samples after Damage

**Figure 8:**
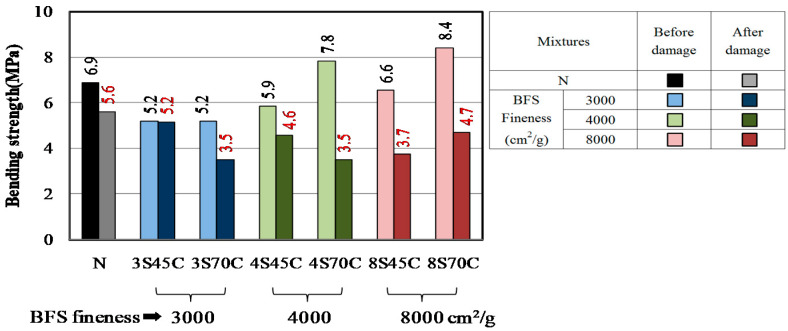
Changes of bending strength in different mortar samples.

**Figure 9:**
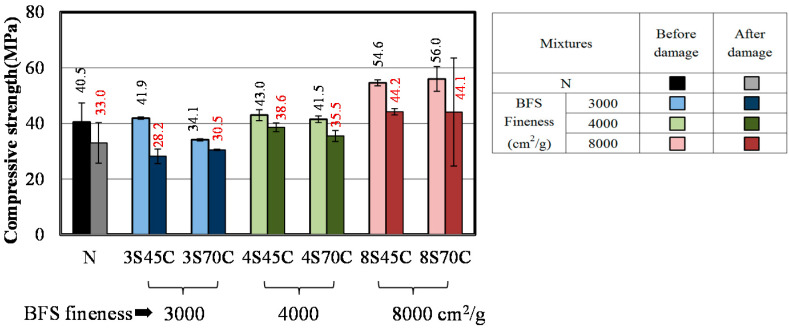
Changes of compressive strength in different mortar samples.

**Figure 10:**
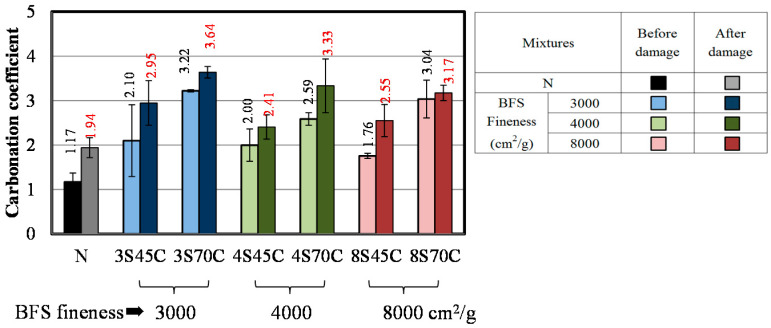
Carbonation coefficients of different mortar samples after damage.

The results for the bending strength and compressive strength of the mortar samples after damage caused by freeze–thaw testing are shown in Figure 8 and Figure 9. Figure 8 shows that the bending strengths of all the samples have decreased after the damage. Similar results are shown in Figure 9. These results indicate that micro cracks were formed in all the mortar samples and increased with the number of freeze–thaw cycles. Figure 10 shows the results of the accelerated carbonation test for the mortar samples. The carbonation coefficients of all the mortar samples after damage are greater than before the damage. The above results clarify that all the mortar samples exhibit decreased bending strength and compressive strength and increased carbonation coefficient due to the micro cracks formed during freeze–thaw cycling [30].

#### 3.2.3. Relative Dynamic Modulus of Elasticity, Bending Strength, Compressive Strength, and Carbonation Coefficient of Mortar Samples after Water Curing

**Figure 11:**
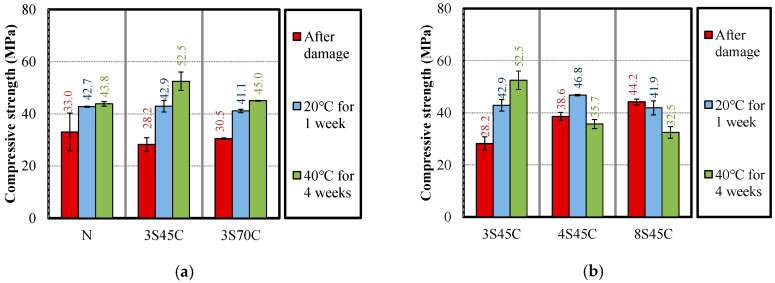
Compressive strength results after damage and after water curing for different (**a**) BFS replacement ratios and (**b**) BFS Blaine fineness values.

As mentioned above, all the mortar samples were immediately stored for 28 days and then damaged to approximately 60% during freeze–thaw testing. Each mortar sample was tested for three cases: after damage, after curing in water at 20 °C for 1 week, and after curing in water at 40 °C for 4 weeks. Figure 11 shows the results of measuring the compressive strength of the mortar samples for different BFS replacement ratios for evaluating the self-healing ability. As shown in Figure 11a, for the sample without BFS, compressive strength recovery was confirmed in the samples cured in water at each temperature; in particular, the 3S45 mortar sample incorporating BFS with lower fineness has a remarkably high strength recovery. This is because the rate of BFS hydration is slow and thus additional reaction occurs after deterioration. Figure 11b presents the effect of increasing BFS replacement ratio on self-healing ability. It can be seen that for the samples 4S45C and 8S45C, the carbonation coefficient decreases with curing in water at 40 °C, but there is no clear explanation for this behavior. The higher Blaine fineness may cause the high temperature-expansion coefficient owing to the hydration of BFS [31], which is needed to investigate the relationship between the self-healing effect of BFS with higher Blaine fineness and the temperature-expansion coefficient.

**Figure 12:**
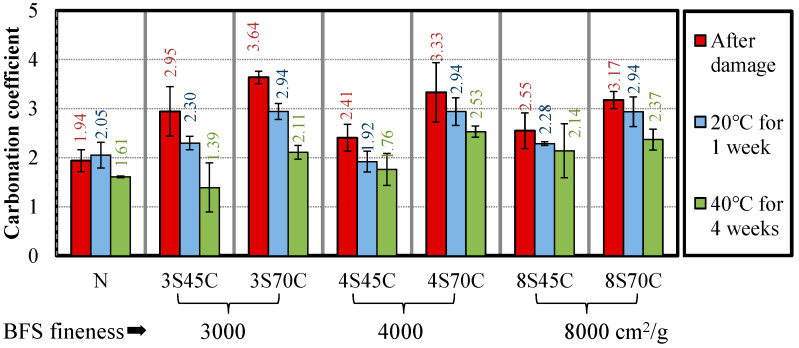
Carbonation coefficients of the mortar samples after damage and after water curing.

Figure 12 shows the results of the accelerated carbonation test for the three stages (after deterioration and after two types of curing condition). After water curing at 20 °C and at 40 °C, the carbonation coefficients of all the mortar samples were decreased after water curing, which was caused by the additional hydration of cement and BFS. In particular, the carbonation coefficient of the mortar sample with BFS was significantly reduced after curing compared to mortar sample N. Therefore, the self-healing effect of BFS, especially with a low fineness of approximately 3000 cm^2^/g, significantly contributes to the decrease of the carbonation coefficient. This may be because the micro cracks become denser as a result of the potential hydration of BFS.

**Figure 13:**
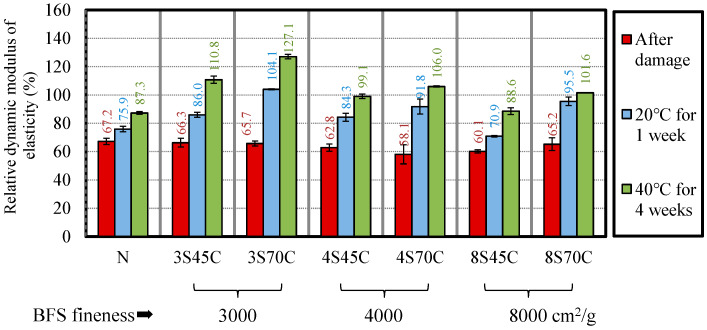
Changes in relative dynamic modulus of elasticity after damage and after water curing.

Figure 13 shows the changes of the relative dynamic modulus of elasticity of the mortar samples. It can be seen from the lower fineness and higher replacement ratio of BFS, especially for 3S45C and 3S70C, that the relative dynamic modulus of elasticity increases in the mortar samples after water curing owing to the remaining products of BFS, implying that incorporating BFS with larger particles in the mixture contributes to the self-healing effect. Matsushita et al. investigated the self-healing properties of concrete using a BFS replacement ratio of 50% and measured the ultrasonic pulse velocity before and after damage [32]. It is reported that the ultrasonic pulse velocity of concrete with BFS after re-curing increased compared to normal concrete.

According to the hydration analysis on the self-healing properties of BFS, Darquennes et al. have studied the early-age self-healing of cementitious materials incorporating BFS under water curing. X-ray tomography test and scanning electronic microscopy were performed. It is reported that volume of healing products of BFS is doubled compared to cement and in new hydration products is mainly C-S-H gel (low CaO/SiO_2_ ratio). Therefore, from Figure 13, increasing the relative dynamic modulus of elasticity due to the presence of new type of C-S-H gel, which generates from the BFS and Ca(OH)_2_ in the BFS mortar sample [33].

#### 3.2.4. Prediction of Self-Healing Performance and Optimum BFS Fineness and Replacement Ratio

In this study, general regression analysis was used to predict the relative dynamic modulus of elasticity of the BFS-blended mortar samples by taking into consideration the Blaine fineness, replacement ratio, and water curing conditions. The regression equation is

**Figure 14:**
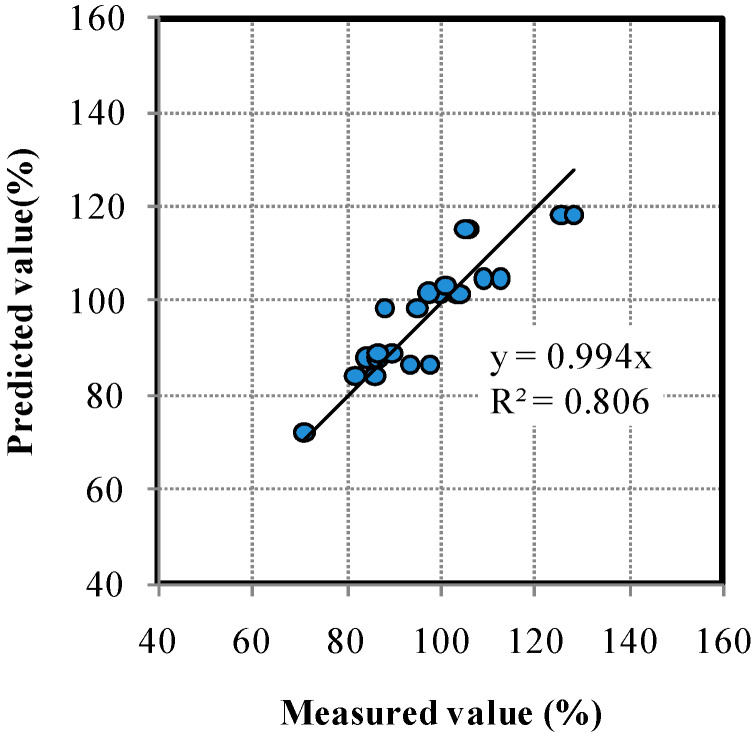
Comparison of the experimental and predicted values of the relative dynamic modulus of elasticity.

Y = 53.7834 − 0.003 × x1 + 0.573 × x2 + 0.8387 × x3(4)

where Y is the calculated relative dynamic modulus of elasticity; x1 is the fineness of BFS, and can be 3000, 4000, or 8000 cm^2^/g; x2 is the BFS replacement ratio; and x3 is the curing temperature in water (20 and 40 °C). Twenty-four mortar samples were used. Table 4 and Figure 14 show the results of general regression analysis. Generally, *p*-value plays a key parameter to evaluate the suggested model. It can be seen that the value of R^2^ was 0.806 and *p*-value was significant at the level of 5% (*p* < 0.05) as shown in Table 5. These values implied that the predicted model can be suitably fitted and can be estimated as 80.6% of variability in the relative dynamic modulus of elasticity between measured and predicted value. The results of a comparison of the experimental results for the relative dynamic modulus of elasticity with the predicted values are presented in Figure 14. The results imply good agreement between the experimental values and the predicted values of the relative dynamic modulus of elasticity.

**Figure 15:**
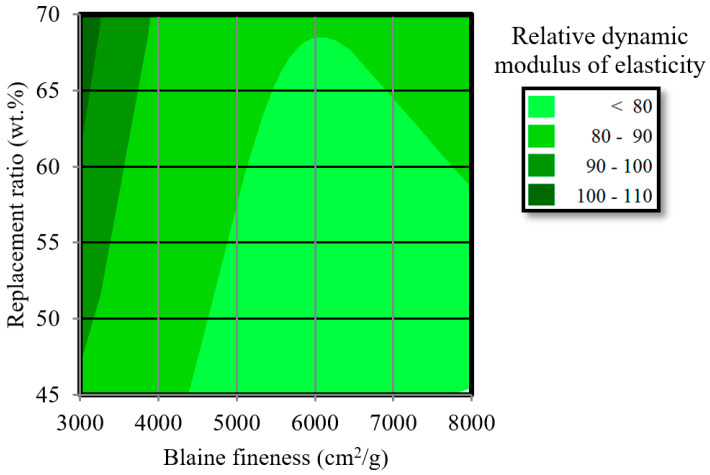
Contour plot of the optimum fineness and replacement ratio for BFS.

Figure 15 shows the optimum Blaine fineness values and replacement ratios for BFS. A contour graph is presented based on the results of the relative dynamic modulus of elasticity. The figure shows that a lower Blaine fineness and a higher replacement ratio for BFS are desirable to achieve self-healing concrete. However, further investigation by taking into consideration the effect of W/B and the presence of CaSO_4_ on the self-healing ability is needed to improve the self-healing mixture proportion to meet design codes for BFS-blended cement.

## 4. Conclusions

The purpose of this study was to investigate the material properties and self-healing ability of mortar incorporating blast furnace slag (BFS), while considering the influence of the replacement ratio and Blaine fineness of the BFS and the curing conditions. The performance of the self-healing ability of mortar samples incorporating BFS was evaluated using mixtures with different replacement ratios, Blaine fineness values, and curing conditions. On the basis of the experimental results, which cover the materials properties and self-healing behavior, the main findings of this study can be summarized as follows:

(1) It was found that replacing cement or sand with BFS in the mortar would increase the bending strength, compressive strength, carbonation coefficient, and durability factor, indicating that the materials properties are dependent on the BFS. The reason for this is the low water-to-binder ratio (W/B) of the BFS-blended cement mixture.
(2) The mortar samples were damaged through freeze–thaw testing until a relative dynamic modulus of elasticity of 60% was achieved. The BFS-blended cement samples may exhibit decreased bending strength, compressive strength, and carbonation coefficient after frost damage. This is because of the micro cracks formed in the mortar during freeze–thaw cycling.
(3) In the investigation of self-healing, it was confirmed that incorporating BFS with a low Blaine fineness and moderate or high replacement ratios (45 or 70 wt %) can improve the compressive strength and relative dynamic modulus of elasticity of the BFS mortar samples. Moreover, these mortar samples displayed higher resistance to carbonation, which was due to the hydration products of BFS.
(4) The relative dynamic modulus of elasticity of the BFS mortar samples after water curing was predicted by a general regression equation (R^2^ = 0.806). Further experimental investigation is needed to improve this equation for a BFS-blended cement mortar mixture. An optimum replacement ratio and Blaine fineness for the BFS was suggested by a contour graph. However, this requires additional investigation regarding the W/B.

## Figures and Tables

**Table 1:** Chemical composition and physical properties of ordinary Portland cement and blast furnace slag (BFS).

Sample	Specific Gravity (g/cm^3^)	Blaine (cm^2^/g)	Chemical Composition (%)							
			SiO_2_	Al_2_O_3_	Fe_2_O_3_	CaO	MgO	K_2_O	Na_2_O	SO_3_
N	3.16	3250	21.5	5.4	2.9	64.3	1.9	0.4	0.2	1.8
BFS3000	2.91	3180	33.8	15.1	0.9	40.8	6.5	–	–	–
BFS4000	2.90	4030	34.1	16.0	0.4	42.1	6.2	–	–	–
BFS8000	2.90	7300	33.6	16.2	0.6	42.1	5.3	–	–	–

**Table 2:** Mixture proportions of all the investigated mortars.

Number	Symbol	W/C	W/B	BFS Blaine Fineness (cm^2^/g)	BFS Replacement		Unit Mass (kg/m^3^)				Flow Value (mm)	Air Content (%)
					Portions	wt %	W	OPC	BFS	S		
1	N	0.55	0.55	-	-	-	342	621	0	1242	258.5	1.6
2	3S45C	0.55	0.55	3000	Cement	45	342	342	280	1242	259.0	1.8
3	3S70C	0.55	0.55	3000	Cement	70	342	186	435	1242	275.0	2.1
4	4S45C	0.55	0.55	4000	Cement	45	342	342	280	1242	237.5	1.4
5	4S70C	0.55	0.55	4000	Cement	70	342	186	435	1242	259.5	1.2
6	8S45C	0.55	0.55	8000	Cement	45	342	342	280	1242	219.5	0.6
7	8S70C	0.55	0.55	8000	Cement	70	342	186	435	1242	224.5	0.3
8	3S20S	0.55	0.45	3000	Sand	20	342	621	124	1118	220.5	0.9
9	3S45S	0.55	0.38	3000	Sand	45	342	621	280	963	219.5	5.0
10	4S20S	0.55	0.45	4000	Sand	20	342	621	124	1118	187.5	0.9
11	4S45S	0.55	0.38	4000	Sand	45	342	621	280	963	187.5	1.1
12	8S20S	0.55	0.45	8000	Sand	20	342	621	124	1118	211.0	1.7
13	8S45S	0.55	0.38	8000	Sand	45	342	621	280	963	154.5	0.9

**Table 3:** Test items for measurement of materials properties.

Mixtures	Bending Strength	Compressive Strength	Accelerated Carbonation	Freezing/Thawing	Mercury Intrusion Porosimetry (MIP)
N	√	√	√	√	√
3S45C	√	√	√	√	√
3S70C	√	√	√	√	
4S45C	√	√	√	√	√
4S70C	√	√	√	√	
8S45C	√	√	√	√	
8S70C	√	√	√	√	√
3S20S	√	√	√	√	
3S45S	√	√		√	
4S20S	√	√	√	√	
4S45S	√	√		√	
8S20S	√	√	√	√	
8S45S	√	√		√	

**Table 4:** Test items for measurement of self-healing properties.

Mixtures	Bending Strength	Compressive Strength	Accelerated Carbonation	Relative Dynamic Modulus of Elasticity
N	√	√	√	√
3S45C	√	√	√	√
3S70C	√	√	√	√
4S45C	√	√	√	√
4S70C	√	√	√	√
8S45C	√	√	√	√
8S70C	√	√	√	√

**Table 5:** Estimated regression coefficients and corresponding *t* and *p*-value from the experimental data.

Parameter Estimate	Coefficient	Standard Error	*t*-Value	*p*-Value
Constant	53.7834	7.64141	7.03841	0.000
Blain fineness	−0.003	0.00059	−5.10849	0.000
Replacement ratio (%)	0.573	0.10116	5.69353	0.000
Curing conditions	0.8387	0.12645	6.63228	0.000
